# Removal of adult subconjunctival Loa loa amongst urban dwellers in Nigeria

**DOI:** 10.1371/journal.pntd.0006920

**Published:** 2018-11-14

**Authors:** Ogugua Ndubuisi Okonkwo, Adekunle Olubola Hassan, Taofik Alarape, Toyin Akanbi, Olufemi Oderinlo, Ayodele Akinye, Idris Oyekunle

**Affiliations:** 1 Department of Ophthalmology, Eye Foundation Hospital Group Eye Foundation Retina Institute, Lagos, Nigeria; 2 Department of Ophthalmology, Eye Foundation Hospital Group, Lagos, Nigeria; Uniformed Services University of the Health Sciences, UNITED STATES

## Abstract

Loiasis is a neglected tropical disease caused by infection with the filarial parasite Loa loa. It is a disease considered by many to be benign. Several reports of trans border importation of the Loa loa worm amongst immigrants and visitors from endemic regions of the world exist. In most cases an adult subconjunctival worm is removed from the patient. An interventional case series is reported and examines the practice of removal of subconjunctival adult Loa loa worms amongst urban dwellers in Nigeria. Four cases of ocular loiasis seen amongst urban dwellers in Nigeria exemplify the different presentations and removal methods of the subconjunctival adult worm. There were 2 males and 2 females aged 35years, 23years, 25years and 30years respectively. Each patient gave a history of having been raised in a rural community in childhood years, during which they were exposed to streams and muddy farm land; and then migrated to the urban community in later years. They all present with the finding of a subconjunctival adult worm, which was successfully removed and identified to be Loa loa. There are more urban dwellers in Nigeria who present with symptoms of foreign body sensation that may be related to the manifestation of a subconjunctival worm and are not recognized. This is because the emphasis on this disease has erstwhile been on the rural, village dwellers and not on urban dwellers. Eye care practitioners working in urban centers need to be aware of the possibility of this presentation, and be ready to remove any subconjunctival worm when it presents.

## Author summary

Subconjunctival adult Loa loa is one of the ocular manifestations of paraisitosis by Loa loa, which is categorized as a neglected benign disease. Loa loa transmission has predominantly been amongst inhabitants in rural communities in Nigeria, one of the countries in the endemic belt of Africa. However we report a case series of 4 patients presenting with subconjunctival Loa loa who in childhood lived in rural communities but later migrated from rural to urban cities where they presented. The varied presentation of the patients in this series and the technique of worm removal are reported.

## Introduction

Loiasis is a neglected tropical disease caused by infection with the filarial parasite Loa loa. It is a disease considered by many to be benign. It is relatively an African disease restricted to the equatorial rain forest regions of Central and West Africa and Nigeria is one of the countries with an endemicity of Loa loa [1,2,3].

The filarial worm migrates in the subcutaneous tissue underneath the skin and is not visible until it migrates to the eye and often appears in the subconjunctival space. It has also been severally reported to occur in the anterior chamber [4,5,6]. When in the subconjuctival space it may be visualized initially by the patient, who may also feel the crawling sensation or by someone else examining the eye. Though Loiasis is regarded as a forgotten disease, not infrequently patients present to general ophthalmic clinics with symptoms of worm like movements in one or both eyes. Commonly, there may be associated symptom of foreign body sensation. In some cases, careful examination may reveal presence of a subconjunctival worm.

Much of the studies and report on L. loa in literature has been in rural communities. In Nigeria fishermen, nomads and farmers have been reported to have significantly higher infection rates than other occupational categories [7]. The African Programme for Onchocerciasis Control (APOC) was launched in 1995 ultimately to eliminate human Onchocerciasis from the African countries in which the disease was endemic [8]. The APOC involved mass dosing of endemic communities with Ivermectin. This program specifically targeted Onchocerca volvulus (responsible for river blindness). It affected microfilaria including Onchocerca volvulus as well as Loa loa. APOC program was mostly implemented in rural communities and villages amongst endemic African countries, however microfilaria persists in several communities and form a reservoir for transmission in the rural community. The desire for improved living standards and quest by the younger working class population for better lifetime opportunities in Nigeria and other African countries has resulted in a population drift of young adults from the rural to the urban centers. It is therefore likely that some of the now urban dwellers may have once lived in rural communities, and may be infected with microfilaria and harbor adult worms as well.

### Ethics statement

Ethical approval for this report was sort from the Eye Foundation Hospital Institution Review Board and was waived as this work involved only a retrospective reporting of information documented in the patients case records. The research was performed according to the principles of the Helsinki declaration and adequate informed consent was obtained from each patient prior to the surgical removal of the subconjunctival worm. Also, a verbal consent to report the case was obtained from the patients through a telephone contact before this case series. This consent was documented in the patient’s case records and was approved by the institution review board. Furthermore, all four subjects were adults.

## Materials and methods

We present retrospectively consecutive cases of subconjunctival adult Loa loa and the removal in four middle age adults who presented in our ophthalmic clinics; to illustrate the diverse patterns of presentation and removal techniques of the subconjunctival worm and to raise the awareness of this forgotten tropical disease now being a presentation in urban and semi urban communities. It should be considered when patients complain of frequent unexplained ocular foreign body sensation.

These four cases offer different perspectives to clinical presentation and illustrate three varied patterns that can be encountered when trying to remove a subconjunctival adult worm. In cases one and four, the life worm was relatively inactive and was easily extracted via a conjunctival incision. In case three, the dead worm was encysted in the subconjunctival space and required detailed dissection of surrounding tissue and wound closure. In case two the actively migrating worm moved away from within view and reach, but after moments of adopting a head and face down position, the worm returned to the superior bulbar conjunctiva and was then removed through a conjunctival incision.

### Case presentation

Case 1: A 35 year old male presented with a complaint of sharp pain in the right eye the previous night lasting few minutes with associated itching and photophobia. There was no previous history of similar complaints. The left eye was normal. Ocular examination revealed; unaided visual acuity of 6/5 both eyes, palpebral conjunctival papillae and mild bulbar conjunctival hyperemia in both eyes. He received topical Olopatadine (a prescription eye drop with mast cell stabilizing and antihistamine effect) for treatment of the presumed ocular allergic condition.

He returned 3 days later with a complaint of a worm moving in the right eye the previous morning. There was no ocular pain or itching and no generalized pruritus, skin rashes, swelling or joint aches. His vision remained unchanged in both eyes, with obvious right eyelid swelling.

**Fig 1:**
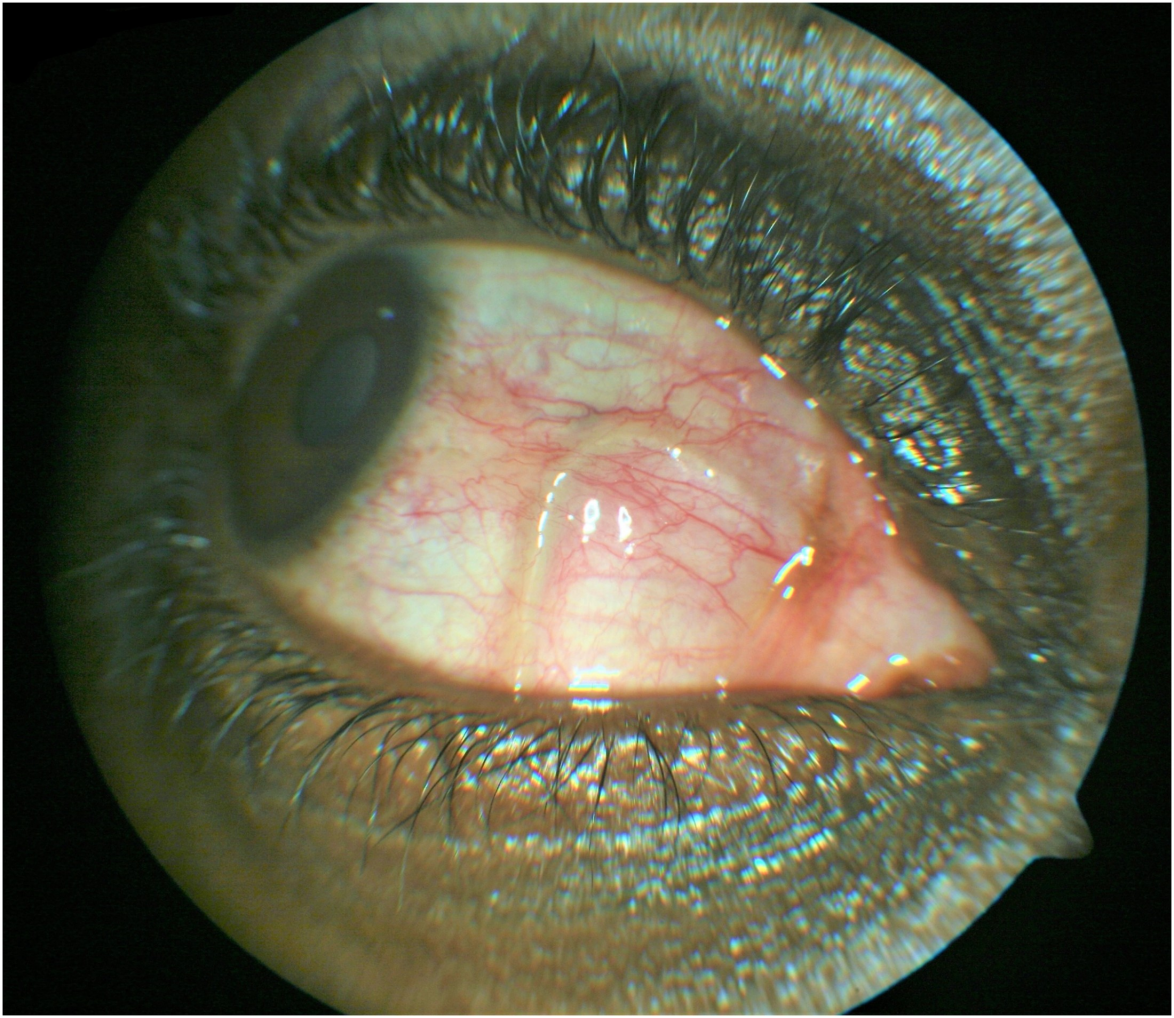
A live adult worm in the nasal subconjunctival space of the right eye.

He had hematological and dermatological investigations, which include; Full Blood Count, peripheral blood film and skin snip test for microfilaria. Results came out as normal, with no eosinophilia noted. However, upon repeat slit lamp examination of the right eye a mobile worm was noticed in the nasal sub conjunctival space "Fig 1". Dilated funduscopy showed cup-to-disc ratio (CDR) 0.3 pink, normal macula, vessels and flat retinae. Systemic examination was normal, no evidence of cutaneous lesions, subcutaneous swellings or nodules. Upon further questioning he gave a history of having worked in swampy rural community farmlands as a child.

**Fig 2:**
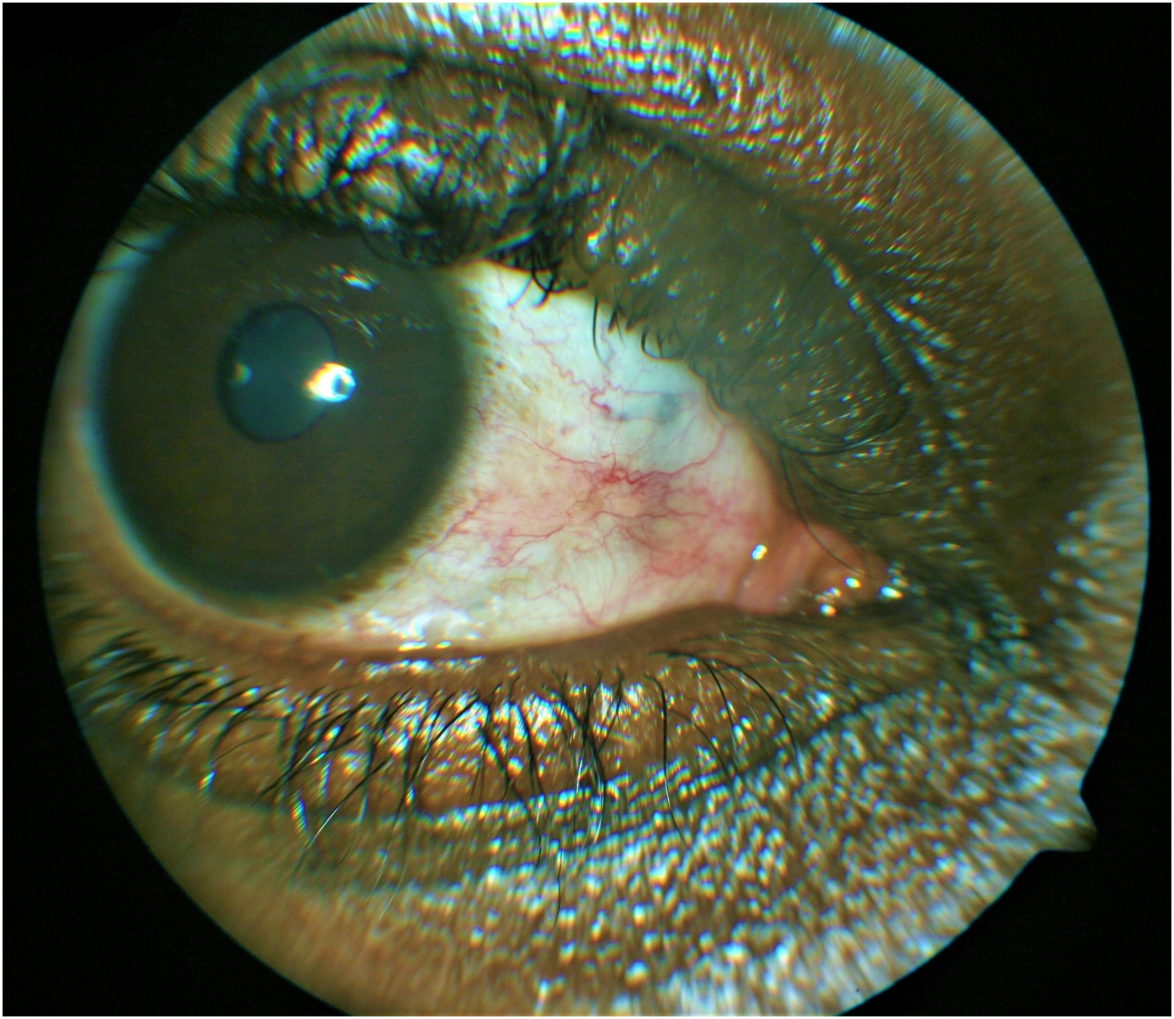
Shows the healed nasal region of the right eye after removal of the worm and healing of the conjunctival incision.

**Fig 3:**
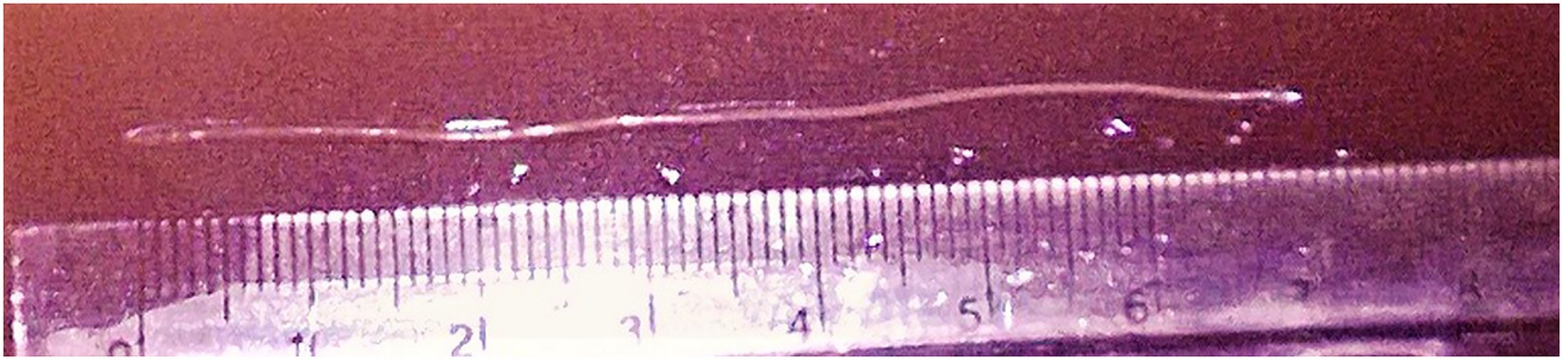
The 7 cm worm extracted from the eye.

He was taken to the operating room the same day and with a retrobulbar anesthesia, a small conjunctival incision was made inferonasally adjacent to the worm. A white colored live worm was grasped with toothless forceps and extracted carefully, intact. Topical antibiotic and steroid preparations were given post surgery. Oral Albendazole was given as therapy targeting any remaining adult worms, and oral Ivermectin targeting microfilaria. Microscopic evaluation of the specimen done at the histopathology laboratory revealed a 7 cm adult male Loa loa worm "Figs 2 and 3".

**Fig 4:**
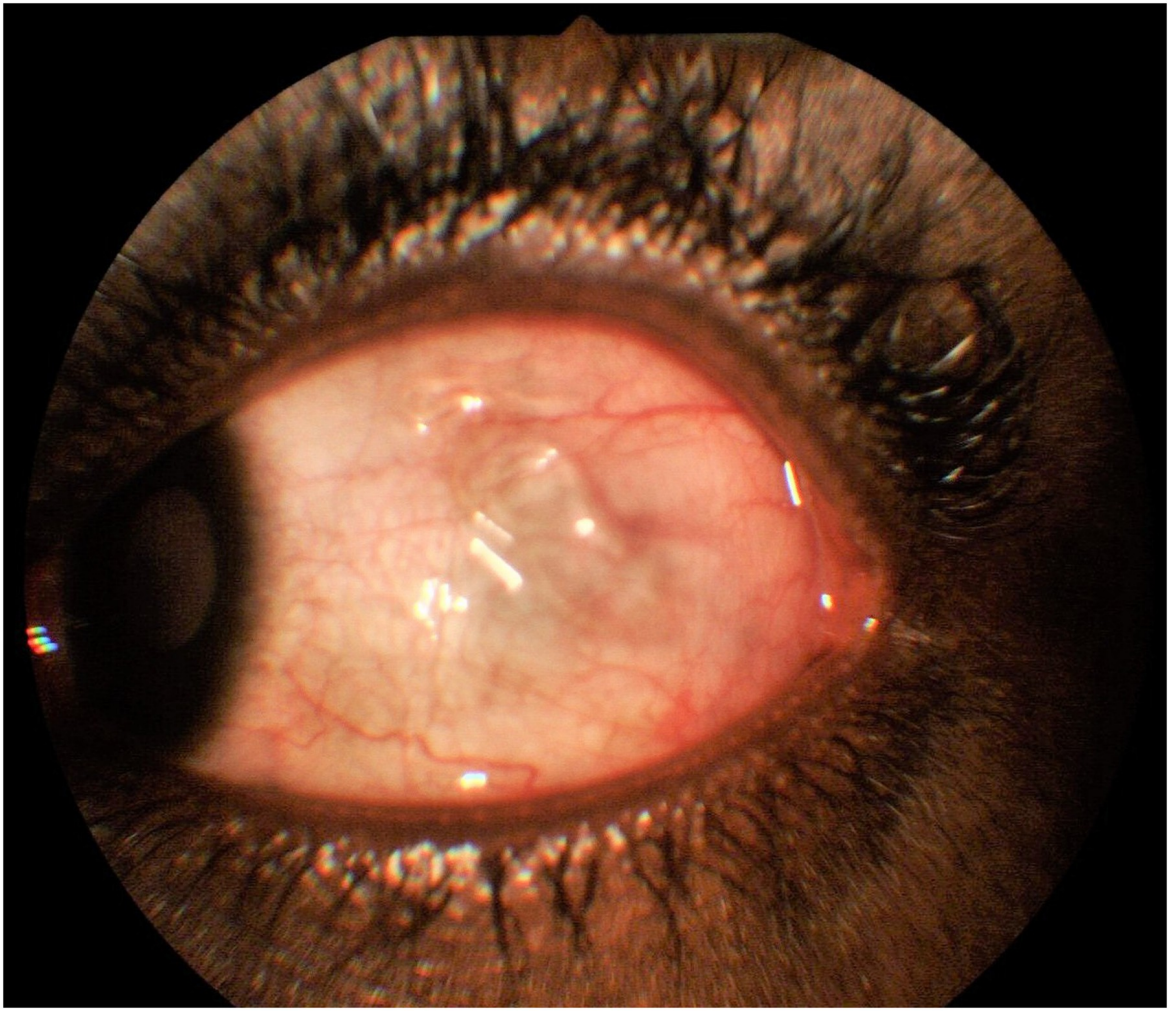
An actively mobile worm in nasal subconjunctival position.

**Fig 5:**
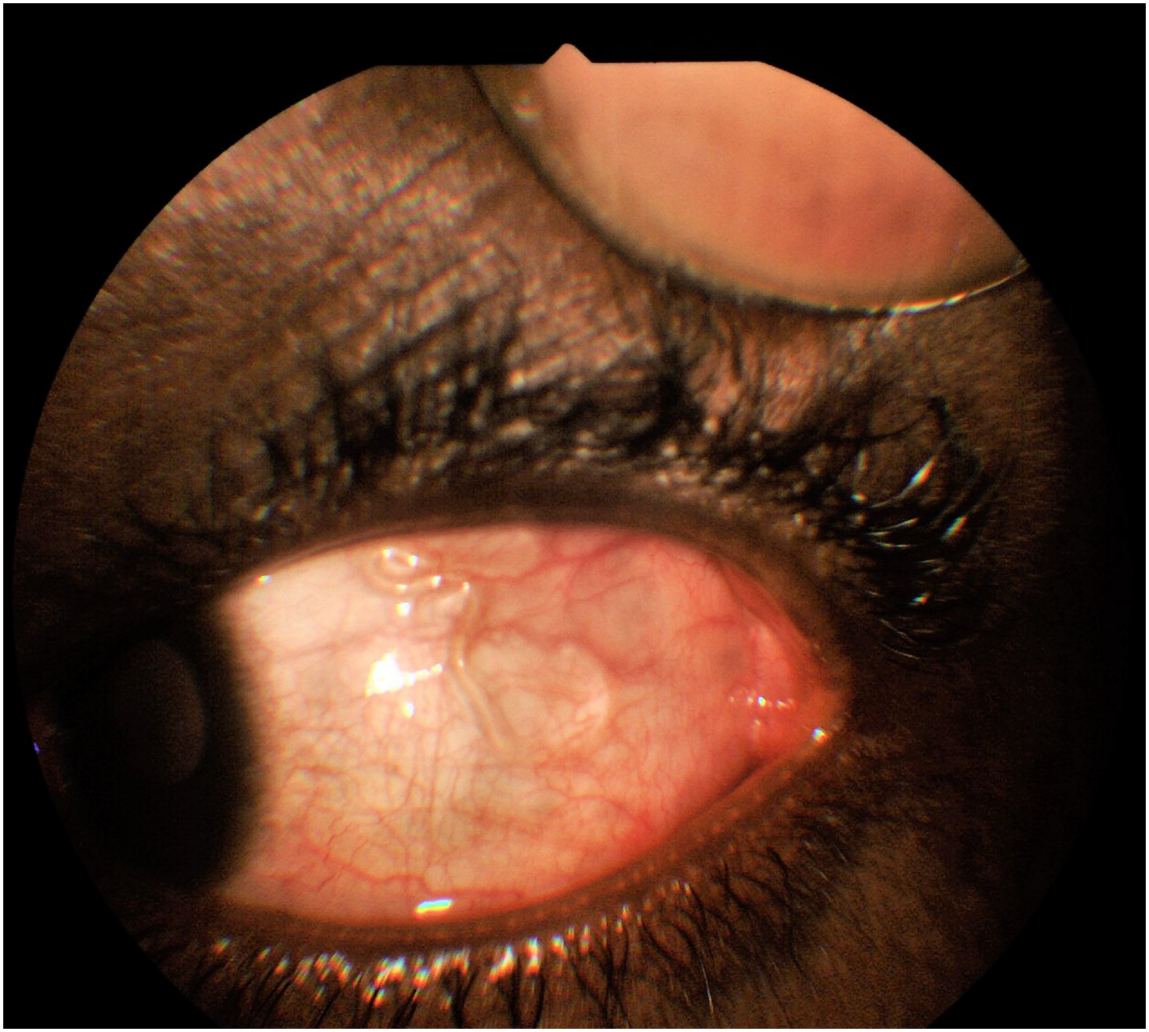
The worm migrating from the nasal position toward the superior fornix and away from view.

Case 2: A 23 year old male, presented with symptoms of crawling sensation and foreign body sensation in his right eye, and had observed a worm in this eye. He had no previous symptoms prior to his presentation. He gave a history of having worked in a cocoa farm plantation during his childhood years and had severally suffered from bites from unknown flies. He had no systemic symptoms nor signs and aside from his ocular complains was healthy. There was no swelling anywhere in the body and no itching. Upon ocular examination his visual acuity was 6/6 in both eyes. The only significant finding was the presence of an actively mobile worm in the nasal subconjunctival space of the right eye "Fig 4". This worm soon migrated upwards towards the superior fornix and away from view during the examination and before removal could be attempted "Fig 5". The patient was immediately asked to adopt a face down position and within 30minutes of this time; he could feel a crawling sensation again in the same eye indicating that the worm was back. He was quickly taken to the operating room and the worm was extracted successfully using a local infiltration of the conjunctiva with lignocaine anesthesia. Histological examination revealed it to be an adult Loa loa worm.

**Fig 6:**
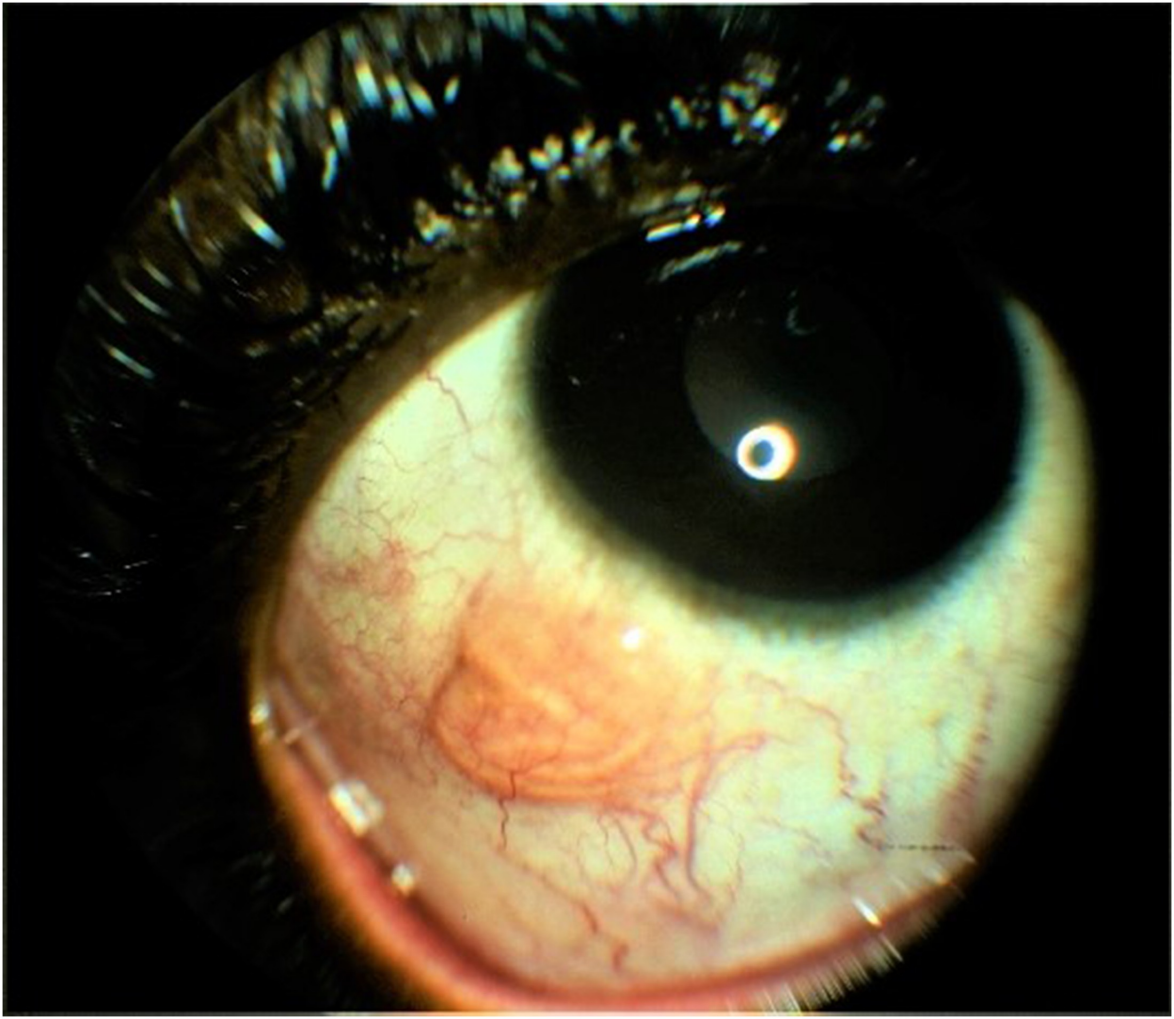
A lifeless encysted worm in the inferior bulbar subconjunctival space.

Cases 3: A 25 year old female who had suffered sensation of movement and foreign body sensation in both eyes for the past 10 years and gave a past history of swimming in rural streams during childhood years. There was no history of swelling on the body and no itching. She had noticed an increasingly frequent occurrence of a worm like movement in both eyes over these years. Following ingestion of diethyl carbamazepine she noticed a sudden appearance of a red patch in the right eye. Upon examination her visual acuity was 6/5 in both eyes. The only significant finding was a localized hyperemic raised lesion on the surface of the right eye. This turned out to be a subconjuctival worm in the inferotemporal subconjunctival space of the right eye. The worm was found to be lifeless and covered by a surrounding cyst wall "Fig 6". Care was taken to dissect the conjunctival and subtenons tissue away from the encysted worm, which was carefully extracted with a toothless forceps. Conjunctival incision site was closed with interrupted sutures. Histology revealed an adult Loa loa worm.

**Fig 7:**
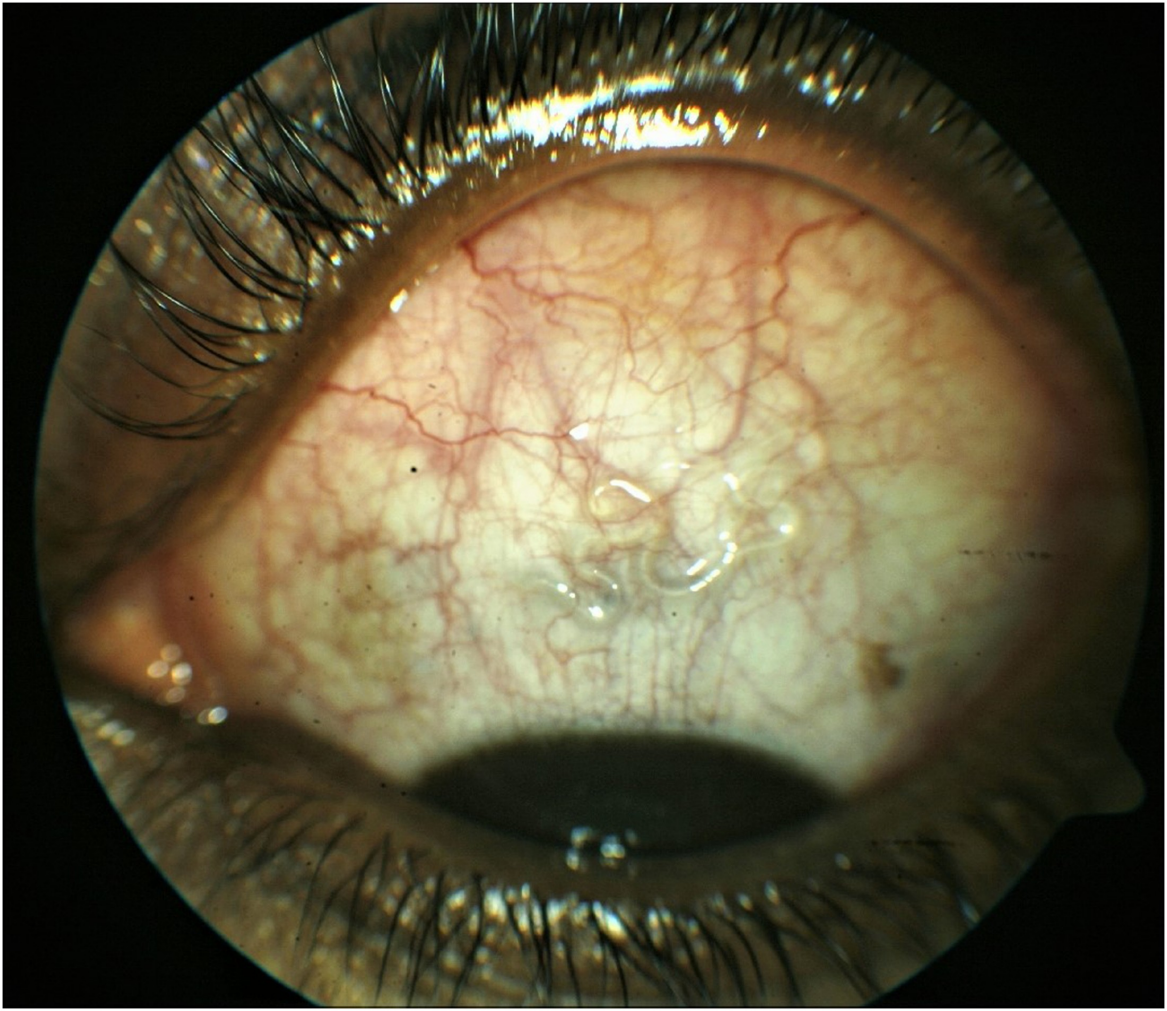
A mobile life worm in the superior bulbar subconjunctival space, which migrated back to the temporal position and was extracted.

Case 4: A 30 year old female who presented with symptoms of redness in the right eye and seeing a worm moving in the left eye. She also gave a history of exposure to rural streams in childhood. On ocular examination, visual acuity was 6/5 in both eyes. She had a temporal subconjunctival hemorrhage as the only ocular finding. The left eye worm she had seen earlier was no longer present. She was reassured and informed to return to the clinic upon seeing the worm again. She re-presented eleven months later upon seeing the worm again, with symptoms of left eye recurrent redness and feeling of something moving in the eye. This was associated with generalized body itching worse at night times. Ocular examination revealed a mobile worm in the temporal subconjunctival area of this eye. The worm soon migrated to the superior bulbar conjunctiva "Fig 7".

Through a conjunctival incision the life worm was extracted using a forceps and the wound was repaired. Histology confirmed an adult Loa loa. Blood work up including investigations for microfilaria was negative; but there was a positive eosinophilia of 44.4%. She was treated with oral Albendazole.

## Results and discussion

Loa loa is a filarial nematode, which infects millions of people in Western and Central Africa, to cause loiasis. It enters the human body through the bite of the deer fly (Chrysops) and explores the subcutaneous tissue of the host undetected, until it enters the subconjunctival space when it becomes symptomatic, visible and presents an opportunity to be physically removed. When present in the subconjunctival space it is freely mobile and the patient complains of a foreign body and, or crawling sensation, seeing a moving worm or tumor, redness and itchy sensation. Its subconjunctival presentation has earned it the name "African Eye Worm." In some cases it can be associated with significant vision loss and other features of uveitis including pain and photophobia when it migrates into the anterior chamber of the eye. Though L. Loa can be seen in the subconjunctival space, anterior chamber and vitreous cavity, it can present with other well known systemic features and can coexist with other parasitic filarial nematodes including; Wuchereria bancrofti and Brugia spp. (lymphatic filariasis), Onchocerca volvulus (Onchocerciasis) and Mansonella spp. (Mansonelliasis)[9].

In 1890 Stephen Mckenzie, an Ophthalmologist, identified microfilaria. In 1895 a localized angioedema was observed in Calabar, a coastal town in Nigeria, resulting in the name “Calabar swelling” by Douglas Argyll-Robertson who was a Scottish Ophthalmologist. Microfilaria of L. loa is transmitted by Chrysops. The two important vectors are Chrysops silacea and Chrysops dimidiata; they prefer rainforest-like environments and only exist in Africa. They are popularly known as deerflies and mango flies [10]. The vectors are blood sucking and day-biting flies; the female fly requires a blood meal for production of a second batch of eggs. Humans are the primary reservoir for L. loa.

Most reported cases of Loiasis are amongst immigrants from Western and Central African countries, and among travelers to this region. L. loa infestation presenting as the "African eye worm" has been well reported as a trans-border disease imported from endemic regions of the world to other non endemic countries. This is seen in several case reports from Europe, Latin America, Asia and the United States of individuals who have imported the parasite by travelling to or living in endemic regions in Central and West Africa [11,12,13,14]. A review of over a hundred infected individuals identified the patients to have made contact with three countries namely Cameroon, Nigeria and Gabon [15]. The disease in a large number of the cases reported, presented as the eye worm, seen in the subconjunctival space.

There have been reports of subconjuctival and intraocular presentation of adult L. loa amongst Nigerians living in urban centers. This is in contrast to expectation and earlier reports of L. loa presenting in rural habitats in Nigeria. Earliest report from Nigeria was by Osuntokun et al in 1975, of the worm in the anterior chamber. Jain et al [16] in 2008; noticed a subconjunctival worm in a 42 year old Nigerian immigrant in Australia, who had no extra-ocular features. Similarly Shah et al in 2010 reported a case of male L. loa in the subconjunctival space of a 21 year old woman who visited Nigeria 6 years earlier [17]. More recent reports in Nigerians have been by Pedro et al [18], Hassan et al [19] and Omolase et al [20]. Our case series adds to the existing numbers.

Loa loa and other filariasis are established diseases seen amongst the villages and rural communities in endemic areas of Nigeria. Several studies have published on the prevalence of this parasitosis in Nigeria [21]. The (APOC) program was specifically targeting rural communities in endemic areas of Africa. This intervention was decisive and judged to have achieved its objectives, having a marked effect on microfilaria including L. loa. Though Loiasis has been regarded as a benign disease, a recent publication demonstrated an association between Loiasis and increased mortality amongst a group of rural Africans [22]. It is therefore not as benign as previously thought. It does appear that awareness of the ocular parasitiosis by L. loa has been greatly reduced. This case series therefore seeks to raise the awareness of its presence in urban and semi urban locations. It is likely that the rural urban drift, which is being experienced in several African countries, will result in patients who have been infected with the parasite in earlier ages but now living in urban centers manifesting the ocular parasitosis. This point is illustrated in all 4 patients who are young educated adults with subconjunctival L. loa, having a past history of living in the rural community at some point in their childhood life. They all gave a history of living in rural community in childhood during which they were exposed to or came in contact with vectors harboring filaria. The 2 males gave a history of working in farmlands at the time of dwelling in the rural community while the 2 females gave a history of exposure to streams.

In most cases, Loiasis is asymptomatic. There have been reports of initial clinical presentation of loiasis from as soon as 5 months after infection and in some cases after 13 years [23]. The life span of an adult worm is unknown, but in some cases may exceed 17years[23]. This long life span ought to be borne in mind when confronted with a case of Loiasis, as the patient may have forgotten or only vaguely recall the history of exposure to the vector.

Loiasis treatment depends on clinical presentation, and is broadly divided into medical using oral drugs including Diethyl Carbamazine(DEC), Albendazole(ALB), Ivermectin(IVM) and surgical; involving removal of the adult worm from the tissue including subconjunctival space and anterior chamber. Surgical removal of the adult worms can be attempted when the worm is within view and antimicrobial can then be used to kill microfilaria and any remaining adult worm. The first successful worm extraction was done in 1778 by Francois Guyot a French surgeon, who removed a worm from a man’s eye among the West African slaves on a French ship to America [24]. A visible subconjunctival L. loa requires removal. This can be safely done by incision of the conjunctiva tissue and extraction of the adult subconjunctival worm with a pair of forceps. This appears to be the commonest situation from several case reports. This was also the situation in the first and the fourth cases in this series and can be safely done with the use of topical anesthesia or local infiltration using an injectable anesthetic agent. The integrity of the adult worm should be preserved for histological examination. If the worm is broken during extraction, it may cause severe local inflammation and provoke a Calabar swelling at any anatomic location. In another case scenario, the subconjunctival L. loa may be dead and encysted in the subconjunctival space. This was seen in case three in which the adult L. loa had died in the subconjunctival space because of the prior ingestion of DEC. This presentation has been previously reported by Lichtinger et al [25] and Carme et al [26] and is a less common presentation. This requires more careful dissection and extraction of the encysted worm without damage for histological review. Case two presents the most challenging situation, in which the adult worm is freely and actively mobile and migrates away from subconjunctival visibility at time of planned removal. In this case the patient was requested to adopt a complete head and face down position and observe a period of waiting. The L. loa worm soon migrated back to visibility in the subconjunctival position.

Two methods of removal of such actively mobile worm have been described and include in one case the adult mobile worm was fixed with a forceps through the conjunctiva, then conjunctival incision and grasping the worm was done with a second forceps in the other hand [27]. In another case the authors describe two consecutive patients with suspected migratory nematodes who were treated promptly by strategic placement of a pharmacological barrier in the forniceal conjunctiva using 1% lidocaine with epinephrine to block the routes of retreat and to immobilize the worms for controlled retrieval [28]. Due to this barrier, the L. loa could be successfully removed without opportunity to retreat.

To conclude, often the presenting complain amongst patients who may eventually be diagnosed as having a subconjunctival worm include a foreign body like sensation, a red eye, itchy eye and a mobile subconjunctival tumor that showed vermiform movements. An un explained complain of such symptoms should raise a clinicians index of suspicion for a subconjunctival worm. Though methods of removal of subconjunctival worm differ, commonly it can be removed with the use of forceps after a conjunctival incision is made. This requires use of topical anesthesia, but other forms of anesthesia such as, local subconjunctival infiltration and retro bulbar anesthesia have been safely and effectively employed. Several reports also exist of removal using a slit lamp bio microscope. In the 4 cases we have reported the removal was done in the operating room.

It is likely that with the rural urban drift and as younger population migrate to the urban areas in search of better living standards in Nigeria and across African this presentation will become more commonly seen by ophthalmologists and eye care providers working in the cities and urban dwellings. Eye care practitioners need to be aware of this and should be prepared to safely extract such worms when they migrate within reach in the subconjunctival position.

## References

[1] Zouré HGM, Wanji S, Noma M, Amazigo UV, Diggle PJ, Tekle AH, et al (2011) The Geographic Distribution of Loa loa in Africa: Results of Large-Scale Implementation of the Rapid Assessment Procedure for Loiasis (RAPLOA). PLoS Negl Trop Dis 5(6): e1210 10.1371/journal.pntd.0001210 21738809 PMC3125145

[2] Kershaw WE, Keay RWJ, Nicholas WL, Zahra A (1953) Studies on the epidemiology of filariasis in West Africa with special reference to the British Cameroons and the Niger delta. IV The incidence of Loa loa and Acanthocheilonema perstans in the rain-forest the forest fringe and the mountain grasslands of the British Cameroons with observations on the species of Chrysops and Culicoides fauna. Ann Trop Med Parasitol 47: 406–425. 13125273 10.1080/00034983.1953.11685587

[3] Amazigo U. The African Programme for Onchocerciasis Control (APOC). Ann Trop Med Parasitol. 2008 9;102, 1:19–22. 18718149 10.1179/136485908X337436

[4] Osuntokun O and Oyin O. “Filarial worm (Loa loa) in the anterior chamber” Brit J Ophthal. (1975) 59: 166 1131358 10.1136/bjo.59.3.166 PMC1017374

[5] Carme B, Kaya-Gandziami G, Pintart D. Localization of the filaria Loa loa in the anterior chamber of the eye. Apropos of a case. Acta Trop. 1984 9;41(3):265–9. 6150622

[6] Barua P, Barua N, Hazarika NK, Das S. Loa loa in the anterior chamber of the eye: a case report. Indian J Med Microbiol. 2005 1;23(1):59–60. 15928426 10.4103/0255-0857.13877

[7] Anosike JC. The status of human filariasis in north-western zone of Bauchi State, Nigeria. Applied Parasitology [01 6 1994, 35(2):133–140] 8087153

[8] Amazigo UV, Obono M, Dadzie KY, Remme J, Jiya J, Ndyomugyenyi R, et al Monitoring community-directed treatment programmes for sustainability: lessons from the African Programme for Onchocerciasis Control (APOC). Ann Trop Med Parasitol. 2002 3;96,1:S75–92 12081253 10.1179/000349802125000664

[9] “Loiasis.” 2009. The Gideon Online. http://web.gideononline.com/web/epidemiology/index.php?disease=11340&country=&view=General.

[10] World Health Organization (WHO). “Vector Control—Horseflies and deerflies (tabanids).” 1997.

[11] Passos RM, Barbosa CP, Almeida Jde S, Ogawa GM, Camargo LM. Subconjunctival Loa loa worm: first case report in Brazil. Arq Bras Oftalmol. 2012 Jan-Feb;75(1):67–70. 22552423 10.1590/s0004-27492012000100015

[12] Bowler GS, Shah AN, Bye LA, Saldana M. Ocular loiasis in London 2008–2009: a case series. Eye (Lond). 2011 3;25(3):389–91. 21242984 10.1038/eye.2010.192 PMC3178318

[13] Carbonez G, Van De Sompel W, Zeyen T. Subconjunctival Loa Loa worm: case report. Bull Soc Belge Ophtalmol. 2002;(283):45–8. 12058486

[14] Verallo O, Fragiotta S, Carnevale C, De Rosa V, Vingolo EM. Subconjunctival Loiasis: case reports and review of cases described in Italy. Clin Ter. 2013;164(2):e127–31. 23698214 10.7417/CT.2013.1544

[15] Antinori S, Schifanella L, Million M, Galimberti L, Ferraris L, Mandia L et al Imported Loa loa filariasis: three cases and a review of cases reported in non-endemic countries in the past 25 years. International Journal of Infectious Diseases 16 (2012) e649–e662 10.1016/j.ijid.2012.05.1023 22784545 10.1016/j.ijid.2012.05.1023

[16] Jain R, Chen JY, Butcher AR, Casson R, Selva D. subconjunctival Loa loa worm. Int J Infect Dis. 2008;12(6):e133- 10.1016/j.ijid.2008.03.001 18434226 10.1016/j.ijid.2008.03.001

[17] Shah A.N., Saldana M. Ocular loiasis. N Engl J Med. 2010;363:71 20848684 10.1056/NEJMicm1002020

[18] Pedro-Egbe CN, Chukwuka IO, Obunge OK. Live adult Loa loa in the anterior chamber of a Nigeria female. Port Harcourt medical journal 2008; 3(1): 104–107.

[19] Hassan S., Isyaku M., Yayo A. Adult Loa loa filarial worm in the anterior chamber of the eye: A first report from savanna belt of Northern Nigeria. PLoS Neglected Trop Dis. 2016;10(4):e0004436. 10.1371/journal.pntd.0004436 PMC4824396 27054771

[20] Omolase CO, Sotiloye OA, Ogunleye OT, Omolase BO. Ocular loiasis in a Nigerian male adult. The N Iraqi J med 2012;8: 86–90.

[21] Ufomadu GO, Nwoke BE, Akoh JI, Sato Y, Ekejindu GO, Uchida A, et al The occurrence of loiasis, mansonellosis and wuchereriasis in the Jarawa River Valley, central Nigeria. Acta Trop. 1990 12;48(2):137–47. 1980569 10.1016/0001-706x(90)90053-3

[22] Chesnais Cédric B, Takougang Innocent, Paguélé Marius, Pion Sébastien D, Boussinesq Michel. Excess mortality associated with loiasis: a retrospective population-based cohort study. The Lancent Infectious Disease. Volume 17, No. 1, p 108–116, 1 2017 10.1016/S1473-3099(16)30405-4 27777031

[23] El- Shabrawi Y. Loiasis In: Foster CS, Vitale AT, Diagnosis and treatment of uveitis. 2nd ed New Delhi: Jaypee Medical Publishers; 2013 pp. 648–655.

[24] Pion S., Chesnais C. (2017) Loiasis In: Marcondes C. (eds) Arthropod Borne Diseases. Springer, Cham

[25] Lichtinger A, Caraza M, Halpert M. Subconjunctival Loiasis. Am J Trop Med Hyg. 2011 2 4; 84(2): 183 10.4269/ajtmh.2011.10-0526 21292882 10.4269/ajtmh.2011.10-0526 PMC3029165

[26] Carme B, Boulesteix J, Boutes H, Puruehnce MF. Five cases of encephalitis duringtreatment of loiasis with diethylcarbamazine. Am J Trop Med Hyg. 1991 6;44(6):684–90.27. 1858969 10.4269/ajtmh.1991.44.684

[27] Sachs HG, Heep M, Gabel VP. Surgical worm extraction in loa loa ophthalmia. Klin Monbl Augenheilkd. 1998 12;213(6):367–9. 10048017 10.1055/s-2008-1035005

[28] Tse BC, Siatkowski R, Tse DT. A technique for capturing migratory periocular worms: a case series and review of literature. Ophthal Plast Reconstr Surg. 2010 Sep-Oct;26(5):323–6. 10.1097/IOP.0b013e3181c563e9 20622701 10.1097/IOP.0b013e3181c563e9

